# Camphor Inhalations in Coryza

**Published:** 1885-01

**Authors:** 


### Camphor Inhalations in Coryza.

Dr. G. E. Dobson writes in the *Lancet:*

This very troublesome complaint has scarcely received the attention it deserves, if we take into consideration the great number of sufferers and the serious laryngeal and pulmonary diseases to which it is too often a prelude. Amongst the host of remedies proposed for its abortive treatmeat, most of which are of doubtful value, or difficult to procure or apply, or even dangerous to use, not one can be named of which it may be said that it is at every one's command, easy of application, unattended with danger, and really effective. No excuse is therefore needed for introducing to the notice of the profession the following simple yet thoroughly effective mode of treatment, which, in my hands, has never disappointed expectations.

About a drachm of camphor, coarsely powdered, or shredded with a knife, is placed in an ordinary shaving jug, which is then half filled with boiling water. The patient having made a paper cone (out of a sheet of brown paper or an old newspaper), large enough to surround his face with the wide extremity, and the mouth of the jug with the other end, proceeds to respire freely, at each inhalation drawing the steam into his nostrils, and at each exhalation forcing it up against the outer surface of his nose and the adjoining parts of his face. A twofold action is produced—the camphorated steam acts internally in a specific manner upon the whole extent of the mucous surfaces, and externally produces perfect diaphoresis of the skin covering the nose and sides of the face, there acting as a derivative from the inflamed Schneiderian membrane.

The jug should be surrounded by a woolen cloth, in order to prevent the water cooling, or better, if a tin can be used, a small spirit lamp or heated iron may be placed beneath it, so as to maintain the heat of the water and the vaporization of the camphor.

The patient should continue his respirations (keeping the margins of the paper funnel closely applied round his face) from ten to twenty minutes, and this should be repeated three or four times in as many hours, till entire freedom from pain is experienced. Great relief is always felt, even after a first application, and three or four usually effect a cure. Camphor, or some of its preparations, have, as is well known, long been in use in the treatment of colds, but the above-described method of employing it in conjunction with the vapor of water, both as an internal and external application at the same time, has not so far as I know, been previously brought to the notice of the profession, or, if brought, has not been recognized in any general or special medical work. The mode of application, however, is all-important, but as this is neither troublesome nor otherwise unpleasant to the patient, nor are the materials difficult to procure, camphor being everywhere a household drug, I believe that those who may give this treatment a trial will find it not only a simple but most effective remedy against coryza.—*The Practitioner.*

